# Bilingualism as a Contributor to Cognitive Reserve? Evidence from Cerebral Glucose Metabolism in Mild Cognitive Impairment and Alzheimer’s Disease

**DOI:** 10.3389/fpsyt.2016.00062

**Published:** 2016-04-15

**Authors:** Magdalena Eva Kowoll, Christina Degen, Lina Gorenc, Anika Küntzelmann, Iven Fellhauer, Frederik Giesel, Uwe Haberkorn, Johannes Schröder

**Affiliations:** ^1^Section for Geriatric Psychiatry, University Clinic Heidelberg, Heidelberg, Germany; ^2^Department of Psychiatry and Psychotherapy, University Hospital Leipzig, Leipzig, Germany; ^3^Department of Nuclear Medicine, University Clinic, Heidelberg, Germany

**Keywords:** bilingualism, cognitive reserve, Alzheimer’s disease, mild cognitive impairment, FDG-PET

## Abstract

**Objective:**

Bilingualism is discussed as one factor contributing to “cognitive reserve” (CR), as it enhances executive control functions. To elucidate the underlying cerebral correlates, regional glucose uptake was compared between bilinguals and monolinguals with mild cognitive impairment (MCI) and beginning-stage Alzheimer’s disease (AD) by using [^18^F]fluorodeoxyglucose (FDG) positron emission tomography (PET).

**Methods:**

Thirty patients (73.2 ± 7.4) diagnosed with MCI or probable AD received physical and neuropsychological examinations, blood tests, and FDG-PET scans. Sixteen patients were classified as lifelong bilinguals, following the criterion of Bialystok et al., and groups were matched for age, sex, and mini mental state examination scores. Analyses were conducted using statistical parametric mapping version 8. The whole brain was used as reference region for intensity normalization and years of education were controlled for.

**Results:**

Bilingual patient groups showed substantially greater impairment of glucose uptake in frontotemporal and parietal regions [including Brodmann areas (BAs) 9, 47, 40, and 21] and in the left cerebellum relative to monolingual patients.

**Conclusion:**

Bilingualism is likely to contribute to CR, given that bilingual patients showed more severe brain changes than monolinguals when adjusting for severity of cognitive impairment. The latter did not only comprise BAs relevant to speech and language but also structures typically involved in AD pathology, such as the temporal and the parietal cortices.

## Introduction

Lifelong bilingualism is associated with higher cognitive reserve (CR), as it is linked to relatively delayed onset of Alzheimer’s disease (AD)-related cognitive deficits (1–4) and the manifestation of mild cognitive impairment (MCI) and AD (4–6). CR facilitates compensation of pathological cerebral changes for a longer period of time; hence, in neuroimaging studies, patients with higher CR typically show more pronounced changes than those with a low CR despite similar levels of impairment. The only study to test this effect in bilinguals was presented by Schweizer et al. (7), who compared indices of brain atrophy using computed tomography (CT) scans of 20 monolingual and 20 bilingual patients diagnosed with probable AD, carefully matched for level of cognitive performance and years of education. Bilingual patients with AD exhibited substantially more pronounced brain atrophy than monolingual patients in indices sensitive to mid temporal changes, specifically the radial width of the temporal horn and the temporal horn ratio.

In the present study, we sought to investigate differences in cerebral glucose metabolism between bilinguals and monolinguals with MCI and AD using [^18^F]fluorodeoxyglucose (FDG) positron emission tomography (PET) under a resting condition, i.e., a neuroimaging technique particularly sensitive to detect AD-related brain changes. We expected to find a substantially greater impairment of glucose uptake in bilinguals than monolinguals.

## Materials and Methods

### Participants

A total of 30 subjects were recruited between June 2012 and March 2014 from the Memory Clinic of the University of Heidelberg. Fourteen subjects were classified as monolinguals, 16 as lifelong bilinguals, following Bialystok et al.’s [p. 460, Ref. (1)] criterion: patients were classified as bilingual if they “… had spent the majority of their lives, at least from early adulthood, regularly using at least two languages,” 12 were diagnosed with MCI, according to the aging-associated cognitive decline criteria [AACD; (8)], and 18 individuals were diagnosed with AD, using the NINCDS–ADRDA criteria (9). Diagnoses were established by consensus between an experienced geriatric psychiatrist and an experienced psychologist.

The bilingual participants consisted of speakers of nine different first languages, of which the most common were German (*N* = 7) and Hungarian (*N* = 2). There were seven different second languages spoken, the most common were German (*N* = 8) and English (*N* = 3). Also, 68.8% (*N* = 11) of bilinguals were multilinguals and were able to use more than two languages. Twelve bilinguals were immigrants to Germany. Their countries of origin were Hungary (*N* = 2), Czechoslovakia, Finland, Palestine, Poland, Peru, Serbia, Slovakia, Taiwan, Trinidad, and Turkey (each *N* = 1).

### Procedure

The study was approved by the Ethical Committee of the University of Heidelberg. After complete description of the study to the participants, informed consent was obtained. Participants were carefully screened for language history, occupational history, fluency in German and other languages, place of birth, and date of immigration.

### Neuropsychological Test Battery

Neuropsychological assessment contained the German version of the CERAD-NP neuropsychological assessment battery (10, 11), the mini-mental state examination (MMSE), the Trail Making Test (TMT) (12), the subtests logical memory and digit span of the German version of Wechsler Memory Scale (WMS-R and WMS-IV) (13, 14), and the clock-drawing test (15). Furthermore, the short version of the Geriatric Depression Scale (GDS) (16) was obtained to exclude depressive symptoms. On the basis of interviews and tests, severity of dementia was rated by using the Global Deterioration Scale (17).

### PET Acquisition Protocol

Following a 6-h fasting, blood glucose level was determined and shown to be below 110 mg/dl in all subjects, before the injection of 118–196 MBq FDG. From 15 min before until 45 min after injection, participants rested in a quiet room with dimmed light. They were instructed to keep their eyes closed. Afterward, 20-min PET scans were acquired. Measurements were obtained with a Biograph 6 by Siemens (thickness of each slice: 5 mm, kVp: 130, pixel size: 0.59 mm × 0.59 mm, and matrix: 512 × 512) [for details, see Ref. (18, 19)].

### Image Analysis

Statistical parametric mapping version 8 (SPM8) routines with default settings were used for basic image processing.^1^ Global normalization was conducted using the proportional scaling option as provided by SPM8. Images were spatially normalized to the Montreal Neurological Institute PET template, written on a matrix with 2 mm × 2 mm × 2 mm voxel size, and smoothed by an isotropic Gaussian filter of 12 mm full width at half maximum [for details, see Ref. (19)].

### Statistical Analyses

For statistical analyses, raw data from the individual CERAD, WMS, and TMT subscores were transformed into *z*-scores that were adjusted for age, gender, and years of education (11–14).

For voxel-based parametric analysis with SPM8, years of education were entered as a covariate. The predicted value was the global normalized glucose uptake. Assuming independency and normal distribution of error terms and homoscedasticity, we performed pairwise one-sided *t*-contrasts for the effect of language group using the contrast matrix (−1, 1) (bilingual < monolingual). Significance level was set to *p* < 0.05 (uncorrected) with cluster extent threshold *k* > 30. The respective structures were identified by their coordinates, according to the Talairach atlas (20) using the Talairach client applet version 2.4.2.^2^ SPSS for Windows version 22 was used for statistical analyses; *p* < 0.05 was considered significant. Likelihood-ratio tests were used where appropriate [for details, see Ref. (19)].

## Results

Demographic and clinical characteristics of the language groups and the total sample are provided in Table 1.

**Table 1:** **Demographic and clinical characteristics of bilingual and monolingual patients with MCI and AD**.

M **±** SD/*N*	Total sample	Bilinguals with MCI and AD (A)	Monolinguals with MCI and AD (B)	*t*-Test/likelihood ratio	
*N*	30	16	14		
Age	73.2 (7.4)	74.6 (6.8)	71.6 (7.9)	*t*(28) = 1.109	
				*p* = 0.277	
♂/♀	14/16	8/8	6/8	LR(1) = 0.153	
				*p* = 0.695	
MMSE	24.4 (2.9)	24.9 (2.7)	23.9 (3.1)	*t*(28) = 0.955	
				*p* = 0.348	
Born in Germany/immigrant to Germany	16/14	4/12	12/2	LR(1) = 11.977***	
				*p* = 0.001	
Years of education	13.6 (4.0)	15.3 (3.6)	11.7 (3.7)	*t*(28) = 2.644*	A > B
				*p* = 0.013	
MCI/AD	12/18	5/11	7/7	LR(3) = 41.455***	
				*p* = 0.000	
Geriatric Depression Scale	2.7 (3.3)	2.7 (3.1)	2.6 (3.7)	*t*(26) = 0.091	
				*p* = 0.928	
Global Deterioration Scale	3.2 (0.7)	3.1 (0.8)	3.4 (0.5)	*t*(28) = −0.932	
				*p* = 0.359	
Clock-drawing test	2.2 (1.1)	2.1 (1.2)	2.4 (1.1)	*t*(28) = −0.552	
				*p* = 0.585	

*LR, likelihood quotient*.

*p < 0.05.

***p ≤ 0.001.

Bilinguals have had more years of education than monolinguals. Moreover, bilinguals were more likely to be immigrants than monolinguals and exhibited a higher proportion of AD pathology as opposed to MCI. Scores on the GDS, the Global Deterioration Scale, and the MMSE showed only minor, non-significant differences between groups (Table 1).

Neuropsychological performance was compared between language groups (Table 2). Overall performance was affected equally in monolinguals and bilinguals. No significant differences occurred.

**Table 2:** ***z*-Scores (means) for the subscales “logical memory” and “digit span” of the Wechsler Memory Scale and TMT and the subscales of CERAD-NP in bilinguals and monolinguals with MCI and AD**.

M **±** SD/*N*	Total sample	Bilinguals with MCI and AD (A)	Monolinguals with MCI and AD (B)	*t*-Test
*N*	30	16	14	
Word list immediate recall	−2.2 (1.2)	−2.0 (1.4)	−2.3 (0.8)	*t*(25) = 0.595
				*p* = 0.557
Word list delayed recall	−1.8 (1.2)	−1.5 (1.3)	−2.2 (1.0)	*t*(25) = 1.534
				*p* = 0.138
Word list recognition	−1.5 (2.3)	−1.5 (2.7)	−1.5 (1.6)	*t*(25) = −0.059
				*p* = 0.954
Constructional praxis	−0.3 (1.6)	−0.1 (1.3)	−0.6 (2.0)	*t*(27) = 0.848
				*p* = 0.404
Constructional praxis recall	−2.1 (1.7)	−2.1 (1.9)	−2.0 (1.3)	*t*(27) = −0.139
				*p* = 0.890
Verbal fluency	−1.3 (1.0)	−1.4 (0.8)	−1.2 (1.3)	*t*(28) = −0.515
				*p* = 0.611
BNT	−0.8 (1.7)	−0.4 (1.4)	−1.2 (1.9)	*t*(28) = 1.446
				*p* = 0.159
TMT-A	−3.3 (4.6)	−3.7 (5.4)	−2.7 (3.7)	*t*(28) = −0.560
				*p* = 0.580
TMT-B	−4.2 (6.4)	−4.0 (7.4)	−4.5 (5.3)	*t*(20) = 0.190
				*p* = 0.851
Logical memory I	−2.1 (1.1)	−2.0 (1.2)	−2.1 (1.1)	*t*(26) = 0.279
				*p* = 0.782
Logical memory II	−2.5 (1.0)	−2.3 (1.0)	−2.7 (0.9)	*t*(26) = 1.015
				*p* = 0.320
Digit span forward	−1.0 (1.0)	−1.0 (1.2)	−1.1 (0.8)	*t*(28) = 0.255
				*p* = 0.801
Digit span backward	−0.9 (1.0)	−0.9 (0.9)	−0.9 (1.0)	*t*(28) = −0.111
				*p* = 0.912

**Figure 1:**
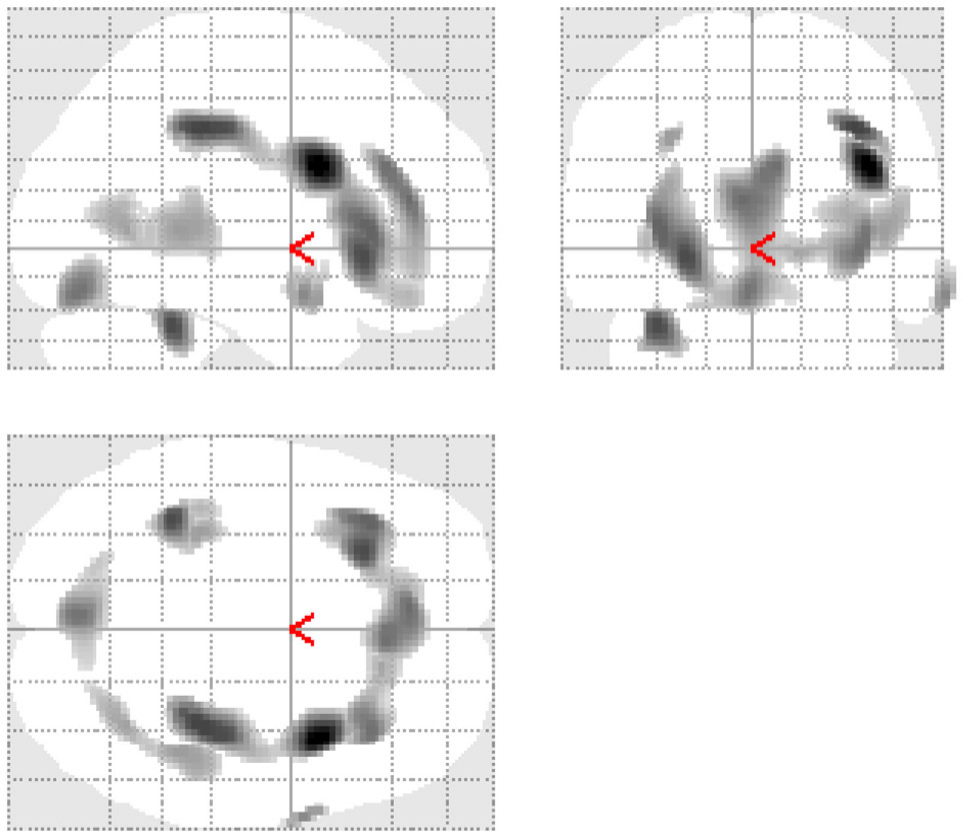
**Glass brains demonstrating significant reduction of glucose metabolism between groups**.

Significant differences in FDG uptake were restricted to lower values in the bilingual vs. monolingual comparison and involved both right and left frontal, temporal and parietal cortices, and the left cerebellum (Figure 1).

As given in Table 3, these regions included Brodmann area (BA) 9, 21, 40, and 47.

**Table 3:** **Reduced glucose metabolism in bilingual MCI and AD patients vs. monolingual MCI and AD patients**.

	Location	BA	Cluster extension	Peak *z-*value	*p*	*x*	*y*	*z*
**R**	**Gyrus frontalis inferior**	**9**	**3357**	**2.56**	**0.005**	**40**	**8**	**26**
R	Inferior parietal lobe	40		2.28	0.011	36	−32	38
L	Gyrus frontalis inferior	47		2.25	0.012	−26	24	−6
**L**	**Cerebellum, culmen**	−	**207**	**2.25**	**0.012**	**−36**	**−44**	−**30**
**L**	**Cerebellum**	−	**441**	**2.08**	**0.019**	**−4**	**−78**	**−18**
L	Cerebellum, declive	−		1.76	0.039	−20	−72	−22
**R**	**Gyrus temporalis medius**	**21**	**51**	**2.00**	**0.023**	**66**	**2**	**−18**
**L**	**Inferior parietal lobe**	**40**	**40**	**1.93**	**0.027**	−**32**	−**34**	**34**
		−	**472**	**1.92**	**0.027**	**44**	−**40**	**4**
		−		1.90	0.029	34	**−**60	4
		−		1.78	0.037	42	**−**52	2
**L**	**Temporal lobe, sub-gyral**	−	**89**	**1.83**	**0.034**	−**40**	−**34**	**0**

*R, right; L, left; BA, Brodmann area*.

*Cluster extension represents the number of contiguous voxel passing the threshold of *p* < 0.05. Bold markings delineate a cluster and the peak *z*-value. Brain regions are indicated by Talairach and Tournoux coordinates, *x*, *y*, and *z*: *x*: the medial to lateral distance relative to the midline (positive: right hemisphere), *y*: the anterior to posterior distance relative to the anterior commissure (positive: anterior), and *z*: the superior to inferior distance relative to the anterior commissure–posterior commissure line (positive: superior) [for details, see Ref. (18)]*.

## Discussion

The present study yielded significantly lower glucose uptake in bilingual compared to monolingual patients with MCI or early AD, although both groups were comparable on clinical grounds. Differences in glucose uptake were localized in areas important for speech and language, mainly involving the frontal cortices as well as in temporoparietal areas traditionally associated with AD pathology (21) and in the left cerebellum. These regions included BA 9 (right), 21 (right), 40 (right and left), and 47 (left). There were no significant differences in neuropsychological domains between monolinguals and bilinguals. These results are in line with the results of Kowoll et al. (22), who showed that bilingual MCI and AD patients showed a similar pattern of neuropsychological deficits as monolingual patients did. This finding also included the TMT-B, which addresses aspects of frontal executive functioning – a domain which seems to be enhanced in healthy bilinguals [(23–25); reviewed in Ref. (26)]. The performance in the BNT – a test in which healthy monolingual subjects usually achieve better scores [(24, 27); reviewed in Ref. (28)] was equally affected in the two language groups in this analysis. Since groups showed only minor, non-significant differences with respect to severity of cognitive deficits, these findings corroborate the hypothesis that bilingualism facilitates compensation of cerebral changes and can thus contribute to CR. That bilinguals also show differences in centers responsible for speech and language processing seems plausible, given the fact that patients differed with respect to language (monolinguals vs. bilinguals) while displaying similar degrees of cognitive impairment.

Brodmann area 9 in the right hemisphere is located in the frontal cortex and contributes to dorsolateral and medial prefrontal cortex functions linked to working memory (29), visuospatial memory (30), and planning (31). BA 21 is located in the middle temporal gyrus, which is involved in language and semantic memory processing (32–35). Likewise, Schröder et al. (21) found BA 21 (both right and left) to be involved in a declarative memory task, using FDG-PET. BA 40 includes Wernicke’s area of the supramarginal gyrus and is functionally involved in reading, meaning, and phonology (36). BA 47 has been implicated in the processing of fine structured stimuli that evolve over time, not merely those that are linguistic (37). Moreover, Dos Santos et al. (38) analyzed neuropsychological deficits with respect to morphometric changes in 94 patients with MCI and AD and found deficits in verbal fluency and word recognition to be significantly correlated with changes in the left gyrus frontalis inferior (BA 47).

Our findings parallel the results reported by Gold et al. (39), who provided the first direct evidence of a neural basis for bilingual cognitive control advantages in aging. The authors compared the reaction times of 80 younger and older adult monolinguals and bilinguals who completed the same perceptual task-switching experiment while functional magnetic resonance imaging (fMRI) was conducted. The researchers observed that bilingual older adults outperformed their monolingual peers with decreased signaling in left lateral frontal and cingulate cortices. This activation increases were directly correlated with enhanced performance on the task-switching task.

Corroborating results of Schweizer et al. (7), our FDG-PET study shows that a group of bilingual patients with AD exhibit substantially greater amounts of brain pathology than monolingual patients when the two groups were matched for level of severity of impairment. Differences in years of education between the two groups were statistically controlled for.

The respective brain regions identified in our study are not only associated with speech and language but also implicated in a number of cognitive functions typically compromised in AD. These findings indicate that bilinguals can compensate for more severe cerebral changes than monolingual patients in the early phases of AD.

## Author Contributions

All authors listed have made substantial, direct, and intellectual contribution to the work, and approved it for publication.

## Conflict of Interest Statement

The authors declare that the research was conducted in the absence of any commercial or financial relationships that could be construed as a potential conflict of interest.

## Footnotes

^1^http://www.fil.ion.ucl.ac.uk/spm/software/spm8/

^2^http://www.talairach.org/index.html
